# Mechanistic insights and environmental ramifications of Cr(III) oxidation to Cr(VI) in soil and groundwater systems: bridging geochemical mechanisms and emerging remediation strategies

**DOI:** 10.1007/s10653-025-02901-2

**Published:** 2025-11-24

**Authors:** Atta Rasool, Eva Pertile, Kateřina Brožová, Jan Halfar, Kristina Čabanová, Petra Malíková, Jitka Chromíková, Oldřich Motyka, Silvie Drabinová, Silvie Heviánková

**Affiliations:** Faculty of Mining and Geology, VSB-Technical University of Ostrava, 17. Listopadu 2172/15, Ostrava-Poruba, 708 00 Czech Republic

**Keywords:** Chromium, Speciation, Mechanism, Risk, Manganese oxides, Mitigation techniques

## Abstract

The oxidation of trivalent chromium [Cr(III)] to hexavalent chromium [Cr(VI)] is a significant environmental process that threatens soil and groundwater quality and poses serious ecological and human health risks. This review provides a critical synthesis of current knowledge about the abiotic, biotic, and thermally induced mechanisms that drive Cr(III) oxidation, including reactions with Mn(IV) oxides, hydrogen peroxide, photochemically generated oxidants, and wildfire-related thermal transformations. Environmental factors such as pH, redox potential, mineralogy, and organic matter content are revealed to play a key role in regulating Cr redox dynamics. Cr(VI) is highly soluble, mobile, and bioavailable**,** exerting toxic effects on microbial communities, plant growth, and human health even at trace levels. Key attenuation pathways, including chemical and microbial reduction, biosorption, and co-precipitation with Fe-bearing minerals, are critically discussed, with particular attention to re-oxidation, which undermines long-term remediation. This review further evaluates emerging remediation strategies**,** such as redox manipulation, biochar-assisted immobilization, and coupled plant–microbe systems, emphasizing the need for site-specific, adaptive approaches that account for spatial and temporal environmental variability. Future research should focus on Cr(VI) generation under post-fire scenarios, isotopic tracing, and field-scale testing of sustainable immobilization materials to improve predictive models and ensure long-term mitigation of Cr(VI) contamination in soil-groundwater systems.

**Supplementary Information:**

The online version contains supplementary material available at 10.1007/s10653-025-02901-2.

## Introduction

Chromium (Cr) is a redox-active transition metal commonly found in ultramafic and serpentine rocks (Liang et al., 2021). In the environment, the two most stable and relevant oxidation states are Cr(III) and Cr(VI) (Ao et al., 2022; Liang et al., 2021). Cr(III) is relatively immobile and acts as a micronutrient in trace amounts, while Cr(VI) is highly soluble, toxic, and carcinogenic (Coetzee et al., 2020; Łożyńska et al., 2025). Its strong mobility in soil and groundwater systems poses serious environmental and human health risks.

Cr contamination has emerged as a global environmental concern due to widespread industrial activities such as electroplating, tanning, metallurgy, and pigment production. Elevated Cr levels have been reported in soils and groundwater across several regions, including tannery-contaminated sites in Kanpur, India; the Hinkley groundwater plume in California, USA; chromite mining areas in Yunnan Province, China; and contaminated aquifers in Cyprus's Ophiolitic complexes (Singh et al., 2019; Zhao et al., 2020). These cases highlight the persistence and mobility of Cr in terrestrial and subsurface environments, particularly the toxic hexavalent form [Cr(VI)], which poses serious ecological and human health risks through groundwater contamination and bioaccumulation. The global scale and severity of such pollution highlight the urgent need to understand the mechanisms that drive Cr(III) oxidation to Cr(VI) and develop effective mitigation and restoration strategies (Kelepertzis et al., 2015; Zhao et al., 2020).

However, various reviews have addressed chromium geochemistry and redox behavior in natural environments (e.g., Guo et al., 2024; Liang et al., 2021), a complete and mechanistic synthesis focusing especially on Cr(III) oxidation to Cr(VI) in soil-groundwater systems is still lacking. This review contributes to current understanding by critically integrating recent findings on both abiotic and biotic pathways, including thermally induced and microbially driven oxidation mechanisms that have not been thoroughly explored in previous studies. It also emphasizes the relationship between mineralogical changes, geochemical factors, and environmental circumstances that influence Cr(VI) production and persistence. Importantly, the review links mechanistic insights to practical implications for risk assessment and remediation, establishing a conceptual framework that integrates laboratory findings with field-scale observations. Collectively, this synthesis provides new views to guide future research and influence the development of effective mitigation measures for Cr(VI) contamination in terrestrial and subsurface ecosystems.

Cr(VI) mainly exists as chromate (CrO₄^2^⁻), dichromate (Cr₂O₇^2^⁻), or hydrogen chromate (HCrO₄⁻), which are readily transported through groundwater and across cell membranes (Kotaś & Stasicka, 2000; Liang et al., 2021). Once inside cells, Cr(VI) is reduced to Cr(III), producing reactive intermediates and reactive oxygen species (ROS) that cause oxidative stress and DNA damage (Singh et al., 2022). In contrast, Cr(III) tends to form insoluble precipitates or adsorb onto minerals and organic matter, limiting its mobility (Dai et al., 2010; Oze et al., 2007).

Although Cr(VI) is traditionally associated with anthropogenic sources, numerous studies have shown that it can also form naturally through the oxidation of Cr(III) under certain environmental conditions (Yakkerimath et al., 2024; Yan et al., 2022). These include reactions with manganese oxides, hydrogen peroxide, and photochemically generated oxidants. Elevated concentrations of geogenic Cr(VI) have been reported in groundwater from China, the United States, and parts of the Mediterranean region, often exceeding drinking water safety limits (Dermatas et al., 2017; Vengosh et al., 2016; Yang et al., 2021).

A further environmental challenge involves the re-oxidation of reduced Cr(III) in remediated soils, sometimes referred to as the “bounce back” effect. Laboratory and field studies have demonstrated that alkaline pH, moisture, and oxygen can facilitate this transformation, thereby undermining remediation efforts and prolonging the persistence of Cr(VI) (Tu et al., 2025; Varadharajan et al., 2017). This phenomenon illustrates the instability of reduced Cr species under fluctuating environmental conditions and highlights the need for a deeper mechanistic understanding of Cr redox cycling. Despite advances in understanding Cr cycling, knowledge gaps remain regarding the kinetics and environmental relevance of Cr(III) oxidation under variable redox conditions (Chen et al., 2024; Zhang et al., 2025). Recent studies have also demonstrated that wildfire temperatures can induce thermal oxidation of Cr(III)-Fe(III) (oxy)hydroxides to Cr(VI) (Burton et al., 2019a, 2019b; Łożyńska et al., 2025), raising concerns in fire-prone regions. These findings indicate that climate-driven and anthropogenic disturbances can interact to promote Cr(VI) production, which has consequences for long-term soil and groundwater quality.

This review provides a comprehensive and mechanistic synthesis of current knowledge on the oxidation of Cr(III) to Cr(VI) in soil-groundwater systems, focusing on both natural and anthropogenically modified environments, including wildfire-affected soils. It critically reviews the abiotic, biotic, and thermally induced processes that drive Cr redox transformations, as well as the impact of major environmental parameters like pH, redox potential, mineralogy, and organic matter. Distinct from previous reviews, this work integrates emerging insights on wildfire-related thermal oxidation and microbially mediated processes, emphasizing their significance under variable redox and temperature conditions. Furthermore, it connects mechanistic understanding to environmental implications and remediation strategies such as chemical reduction, biosorption, and mineral-based immobilization. The paper is structured to systematically address Cr occurrence and speciation, oxidation mechanisms, controlling factors, environmental implications, and remediation strategies. By combining mechanistic depth with applied relevance, this review provides an integrated framework to advance understanding of Cr redox dynamics and guide sustainable management of Cr(VI) contamination in soils and groundwater.

## Sources and occurrence of Cr(III) and Cr(VI)

### Geogenic and anthropogenic origins

**Fig. 1:**
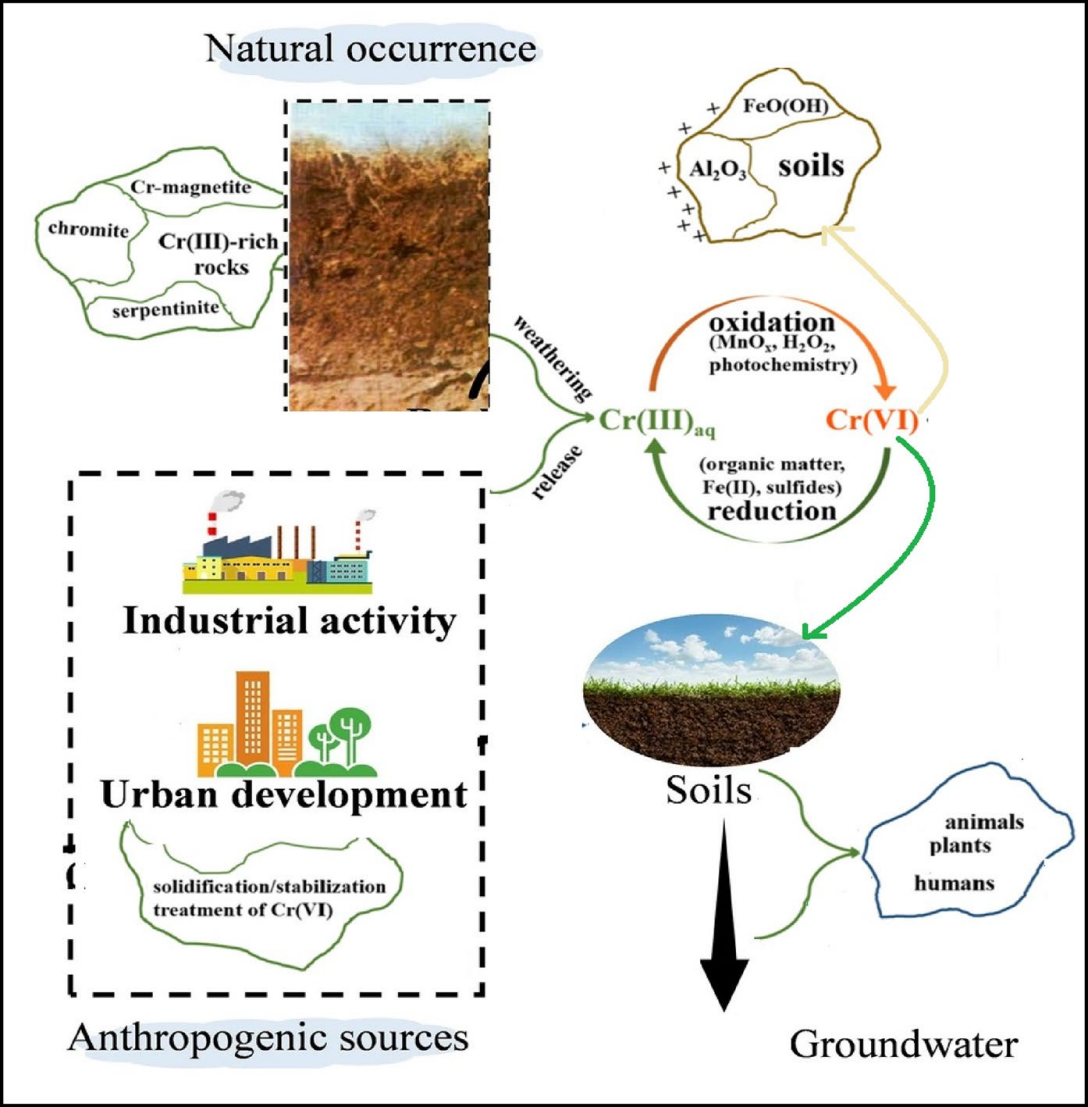
Environmental cycling of chromium, illustrating both geogenic and anthropogenic sources, redox transformations between Cr(III) and the toxic Cr(VI), and its mobility in soil and groundwater

Chromium occurs naturally in ultramafic and mafic rocks such as peridotite, serpentinite, and chromitite (Fig. 1, Table 1), where Cr(III) is present in minerals like chromite (FeCr₂O₄), Cr-substituted magnetite, and silicates including olivine and serpentine (Hseu & Iizuka, 2013; Liang et al., 2021; Oze et al., 2007). Weathering of these rocks releases Cr(III) into soils, where it typically sorbs to clay minerals or co-precipitates with Fe/Al (hydr)oxides, limiting mobility (Tu et al., 2025). Under well-aerated, alkaline conditions, Cr(III) can be oxidized to Cr(VI) by Mn(IV) oxides, especially in serpentine soils and ultramafic terrains (Aiken et al., 2023; Hausladen & Fendorf, 2017). Due to its high solubility and weak sorption, Cr(VI) is highly mobile and toxic (Becquer et al., 2003).

**Table 1:** Geogenic and anthropogenic sources and environmental occurrence of Cr(III) and Cr(VI)

Chromium Form	Source Type	Sources	Occurrence/Form	References
Cr(III)	Geogenic	Weathering of chromite, a natural mineral	Cr^3^⁺ ions, Cr(OH)₃, CrₓFe₁₋ₓ(OH)₃ hydroxides	(Aharchaou et al., 2022)
	Anthropogenic	Textile dyeing, ceramics, photography, glass, tanning, plating, ore processing	Cr^3^⁺ ions, Cr(OH)₃, CrₓFe₁₋ₓ(OH)₃; also from Cr(VI) reduction	(Jagupilla et al., 2015; Wu et al., 2015)
Cr(VI)	Geogenic	Cr(III) oxidation in soils/sediments; fire events	Soluble CrO₄^2^⁻, Cr₂O₇^2^⁻; Cr(III)-Fe(III)-(oxy)hydroxides	(Burton et al., 2019a, 2019b; Hua et al., 2024)
	Anthropogenic	Chromate production, stainless steel, tanning, plating, dyeing, pigments	Soluble CrO₄^2^⁻ and Cr₂O₇^2^⁻; key industrial contaminants	(Dhal et al., 2013)

Anthropogenic sources of Cr include pigment production, electroplating, leather tanning, and chromite processing, which primarily release bioavailable Cr(VI) oxyanions (Dhal et al., 2013; Jobby et al., 2018). Cr(III) is also discharged by textile and glass industries and exists as Cr^3^⁺ ions, Cr(OH)₃ precipitates, mixed Cr-Fe hydroxides, and organic/inorganic complexes (Krüger et al., 2017; Liang et al., 2021). Some of these forms may re-oxidize to Cr(VI) under thermal or oxidative conditions, such as in wildfires or remediated soils (Burton et al., 2019a, 2019b; Jagupilla et al., 2015). Remediation approaches, such as stabilization with Fe^2^⁺ or sodium dithionite, reduce Cr(VI) to less mobile Cr(III) compounds (Wu et al., 2022).

### Chromium forms in soils and sediments

In soils, chromium predominantly exists as Cr(III) and Cr(VI), whose distribution depends on redox conditions. Cr(III) is stable in reducing environments and has low mobility due to strong sorption onto clay minerals, organic matter, and Fe/Mn (hydr)oxides, or structural incorporation into secondary minerals like goethite and ferrihydrite (Liang et al., 2021; Rajapaksha et al., 2013). In contrast, Cr(VI) is stable under oxidizing and alkaline conditions, occurring as soluble and bioavailable oxyanions (Apte et al., 2006). Environmental factors, including pH, redox potential, mineralogy, organic ligands, and competing ions, influence chromium speciation.

### Occurrence in groundwater and surface waters

Chromium occurs in aquatic environments primarily as Cr(III) and Cr(VI), with speciation influenced by redox conditions, pH, mineralogy, and the presence of organic matter. Cr(III) is prevalent under reducing conditions and tends to adsorb onto sediments, whereas Cr(VI) is stable under oxidizing, alkaline conditions and is highly soluble, mobile, and toxic (Baraud et al., 2017; Izbicki et al., 2015). Cr(VI) frequently dominates in oxic groundwater near ultramafic rocks, where Mn oxides and high pH enhance geogenic oxidation of Cr(III) (Ning et al., 2025).

Natural groundwater typically contains < 10 µg/L Cr(VI) (Table S1), though levels up to 300 µg/L have been reported in ultramafic aquifers in China and Cyprus due to oxidizing conditions and mineral weathering (Ning et al., 2025; Zissimos et al., 2021). In California, 31% of sampled wells contained detectable Cr(VI), with 4% exceeding the 10 µg/L MCL (Izbicki et al., 2015). Surface waters often exhibit lower Cr(VI) levels (< 5 µg/L) due to dilution, unless affected by industrial discharge (Kaprara et al., 2015).

Cr(VI) mobility is enhanced in alkaline, low-clay systems with prolonged water–rock interaction, especially under oxic conditions (Morrison et al., 2015; Xu et al., 2021). In contrast, reducing conditions, organic matter, Fe^2^⁺, and sulfides facilitate the reduction of Cr (VI) to Cr(III) (Boussouga et al., 2023; Eckbo et al., 2022). Microbial reduction (e.g., by *Pseudomonas, Shewanella*) also affects Cr(VI) persistence in anoxic environments (Zhan et al., 2023). Land use has a strong influence on mobility, as agricultural and urban areas promote Cr(VI) transport through recharge, nitrate-driven redox shifts, and anion competition (Fu et al., 2025; Wang et al., 2020). These interactions underscore the importance of monitoring Cr speciation for water quality and risk assessment.

## Cr (III) oxidation mechanisms

**Fig. 2:**
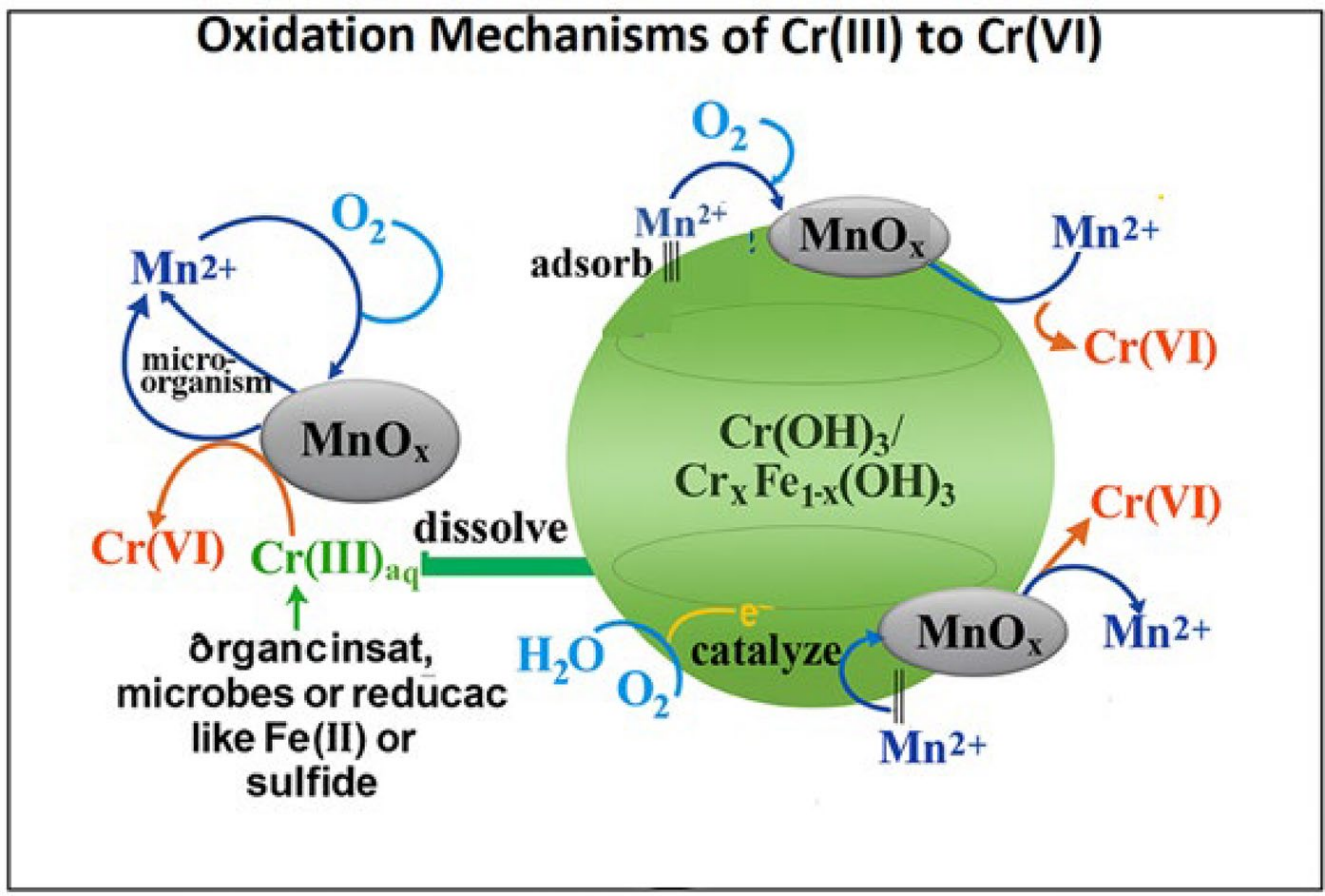
The mechanisms of Cr(III) oxidation by MnOx: dissolved oxidation; adsorbed oxidation; and catalyzed oxidation (Liang et al., 2021)

The conversion of Cr(III) to Cr(VI) in soils and groundwater is a multifaceted process driven by various environmental and biogeochemical conditions (Fig. 2, Table 2). Although Cr(III) is less toxic and more stable than its hexavalent equivalent, it can be converted to Cr(VI) under aerobic and acidic circumstances, particularly in the presence of naturally occurring oxidants (Liang et al., 2021). Understanding the mechanisms that govern Cr(III) oxidation is crucial for assessing the potential mobility and bioavailability of chromium species in the environment, particularly in areas affected by both natural and anthropogenic sources (Liang et al., 2021; Zheng et al., 2020).

**Table 2:** Mechanisms of Cr(III) Oxidation to Cr(VI)

Mechanism	Key Reactants/Conditions	Favorable Conditions	Reference
Mn Oxidation	MnO₂, other Mn oxides	Aerobic, pH 5–9, high Mn	(Liang et al., 2021)
H₂O₂ Pathway	H₂O₂ (abiotic/microbial), Cr(OH)₃, Mn^2^⁺ catalyst	Anaerobic, alkaline aquifers	(Tang et al., 2014)
Photochemical Oxidation	UV, Fe(OH)^2^⁺, organics, OH•	Sunlit soils, low DOC	(Lotfi-Kalahroodi et al., 2023)
Fire-Induced Oxidation	200–800 °C, Cr–Fe (oxy)hydroxides	Wildfires, controlled burns	(Burton et al., 2019a, 2019b)

### Cr (III) oxidation by manganese oxides

Manganese oxides (MnOₓ) such as birnessite, pyrolusite, and vernadite, are key natural oxidants of Cr(III), particularly in well-aerated soils and sediment–water interface (Aiken et al., 2023; Zhang et al., 2025). These minerals oxidize Cr(III) through direct electron transfer at the solid-solution interface (Fig. 2), with the efficiency strongly dependent on environmental pH. At acidic to near-neutral pH levels, Mn(IV) oxides, such as δ-MnO₂, are highly reactive. However, at higher pH values, MnOₓ surfaces may become passivated due to precipitation or carbonate complexation, thereby reducing their redox activity (Chen et al., 2021).

Cr(III) oxidation typically occurs via adsorption onto MnOₓ surfaces followed by surface-mediated electron transfer. Several reactions (Eqs. 1–5) demonstrate the formation of Cr(VI) species (e.g., HCrO₄⁻) with Mn^2^⁺ as a byproduct. Mn(II), while not an oxidant itself, can be reoxidized in the environment to regenerate Mn oxides, contributing to redox cycling (Chen et al., 2021).

1 $$Cr\left( {OH} \right)^{2 + } \, + \, 1.5 \, \delta - MnO_{2} \, \left( s \right) \, \to \, HCrO_{4}^{ - } \, + \, 1.5 \, Mn^{2 + }$$

2 $$Cr\left( {OH} \right)^{2 + } \, + \, 3 \, \beta - MnO_{2} \left( s \right) \, + \, 3 \, H_{2} O \, \to \, HCrO_{4}^{ - } \, + \, 3 \, MnOOH\left( s \right) \, + \, 3 \, H^{ + }$$

3 $$Cr\left( {OH} \right)^{2 + } \, + \, 1.5 \, \delta - MnO_{2} \left( s \right) \, \to \, HCrO_{4}^{ - } + \, 1.5 \, Mn^{2 + }$$

4 $$Cr\left( {OH} \right)^{2 + } \, + \, 3 \, MnOOH\left( s \right) \, + \, 3 \, H^{ + } \, \to \, HCrO_{4}^{ - } \, + \, 3 \, Mn^{2 + } \, + \, 3 \, H_{2} O$$

5 $$Cr^{3 + } \, + \, 1.5 \, \delta - MnO_{2} \, \left( s \right) \, + \, H_{2} O \, \to \, HCrO_{4}^{ - } \, + \, 1.5 \, Mn^{2 + } \, + \, H^{ + }$$

Organic ligands, mineral surface area, and crystallinity of MnOₓ phases all modulate the reaction rate. In natural systems, Mn-bearing minerals such as birnessite and hausmannite support these processes, especially in oxic or seasonally oxidized soils. Although Mn(II)-mediated Cr(III) oxidation is slower, it remains environmentally relevant under fluctuating redox conditions (Aiken et al., 2023; Chen et al., 2021).

### Cr (III) oxidation via hydrogen peroxide

In suboxic to anoxic environments, hydrogen peroxide (H_2_O_2_) can act as a key oxidant of Cr(III) (Li et al., 2022; Luo & Chatterjee, 2010), especially when molecular oxygen is limited (Fig. 2). H_2_O_2_ may form through geochemical processes (e.g., serpentinization) (Foustoukos et al., 2011), microbial metabolism (Luo & Chatterjee, 2010), or photochemical reactions involving dissolved organic matter (Xie et al., 2024).

Under alkaline conditions (pH > 7.5), H_2_O_2_ directly oxidizes Cr^3^⁺ to chromate (CrO₄^2^⁻) via a base-catalyzed two-electron transfer (Luo & Chatterjee, 2010), as shown in Eq. 6. H₂O₂ can also act indirectly via Fenton-like reactions, reacting with Mn(II) or Fe(II) to generate reactive oxygen species (ROS), especially hydroxyl radicals (•OH), which are powerful oxidants of Cr(III) (Djellabi et al., 2022; Sun et al., 2024). This pathway is particularly relevant in subsurface systems with limited O₂ but abundant Mn^2^⁺ and microbial activity.

6 $$3 \, H_{2} O_{2} \, + \, 2 \, Cr^{3 + } \, + \, 10 \, OH^{ - } \, \to \, 2 \, CrO_{4}^{2 - } \, + \, 8 \, H_{2} O \, \left( {pH > 7.5} \right)$$

Equation 7 highlights Mn^2^⁺-driven production of •OH via H₂O₂, contributing to Cr(III) oxidation. Although Mn^3^⁺ and •OH are short-lived, their high reactivity enables significant Cr(VI) generation under favorable redox conditions. The presence of Fe/Mn minerals and precursor silicates such as olivine or pyroxenes enhances H₂O₂ production and stability, especially in alkaline aquifers (Jiang et al., 2024; Zeng et al., 2023).

7 $$H_{2} O_{2} \, + \, Mn^{2 + } \, \to \, Mn^{3 + } \, + \, \bullet OH \, + \, OH^{ - } \, \left( {pH \, > 7} \right)$$

Overall, H₂O₂-driven Cr(III) oxidation represents an important non-oxygen pathway for Cr(VI) formation, particularly in reducing environments influenced by microbial redox activity or weathering of ultramafic rocks.

### Photochemical oxidation of Cr (III)

Photochemical oxidation is a vital surface process for Cr(III) transformation, particularly in sunlit soils and aquatic systems (Fig. 2). UV light can promote the generation of reactive oxygen species (ROS), such as hydroxyl radicals (•OH), through photoreduction of Fe(III) and Cr(III) complexes (Zhang et al., 2024). These ROS facilitate the oxidation of Cr(III) to its hexavalent form Cr(VI), increasing its mobility and toxicity.

Cr(III) commonly forms complexes with hydroxides, iron oxides, or organic matter in soils. Under UV exposure, especially in dry or semi-arid regions, these complexes undergo photochemical reactions that enhance Cr(VI) production (Chen et al., 2021; Hao et al., 2022). The mineral form and complexation state of Cr(III) significantly affect its photo-oxidation potential Cr(OH)₃ and Cr-humic complexes are more reactive than stable forms like chromite (Liang et al., 2021).

Equations 8 and 9 illustrate this process: Fe(OH)^2^⁺ undergoes photolysis to release •OH (Eq. 8), which in turn oxidizes Cr^3^⁺ to CrO₄^2^⁻ (Eq. 9). These reactions are relevant to shallow environments where iron and chromium coexist, particularly under high solar irradiance (Zhang et al., 2024; Zulfiqar et al., 2023).

8 $$Fe\left( {OH} \right){}^{2 + } \, + \, h\nu \, \to \, Fe{}^{2 + } \, + \, \bullet OH$$

9 $$Cr^{3 + } \, + \, \bullet OH \, \to \, Cr^{6 + } \, + \, OH^{ - }$$

### Redox cycling and environmental dynamics of Cr(III)/(VI)

A dynamic redox cycle between Cr(III) and Cr(VI) may occur in redox-active soils and sediments, especially in environments with fluctuating oxygen levels such as wetlands or seasonally flooded zones (Izbicki et al., 2015; McClain et al., 2019). Under anaerobic conditions, Cr(VI) can be reduced to Cr(III), while reoxidation may occur when oxic conditions return.

Microbial activity plays a central role in this redox cycling (Fig. 2), influencing both Cr(VI) reduction and Cr(III) oxidation through reactive intermediates or by modulating local redox conditions (Rahman & Thomas, 2021). The distribution and persistence of chromium species are thus closely tied to environmental dynamics and the function of microbial communities.

UV-induced transformations may also contribute. In Eq. 10, UV light photoreduces [Cr(OH)]^3^⁻ⁿ to Cr^2^⁺ and hydroxyl radicals (•OH), and in Eq. 11, Cr^2^⁺ is reoxidized to CrO₄^2^⁻ in the presence of O₂ and OH⁻ ( Ceballos et al., 2023; Liang et al., 2021). These pathways highlight how transient species can bridge the Cr(III)/Cr(VI) cycle under surface conditions.

10 $$\left[ {Cr\left( {OH} \right)} \right]^{3 - n} \, + \, h\nu \, \to \, Cr^{2 + } \, + \, \bullet OH\frac{x - \mu }{\sigma }$$

11 $$Cr^{2 + } \, + \, O_{2} \, + \, 4 \, OH^{ - } \, \to \, CrO_{4}^{2 - } \, + \, 2 \, H_{2} O$$

Organic carbon, clay minerals, and iron/manganese oxides further influence Cr(VI) mobility by modifying sorption, redox interactions, and transport behavior (Cao et al., 2021; Ramli et al., 2023). In organic-rich soils, microbial reduction of Cr(VI) may limit its long-term mobility and toxicity.

### Natural and fire-induced Cr(VI) formation from Cr(III)-Fe(III)-(Oxy)hydroxides

**Fig. 3:**
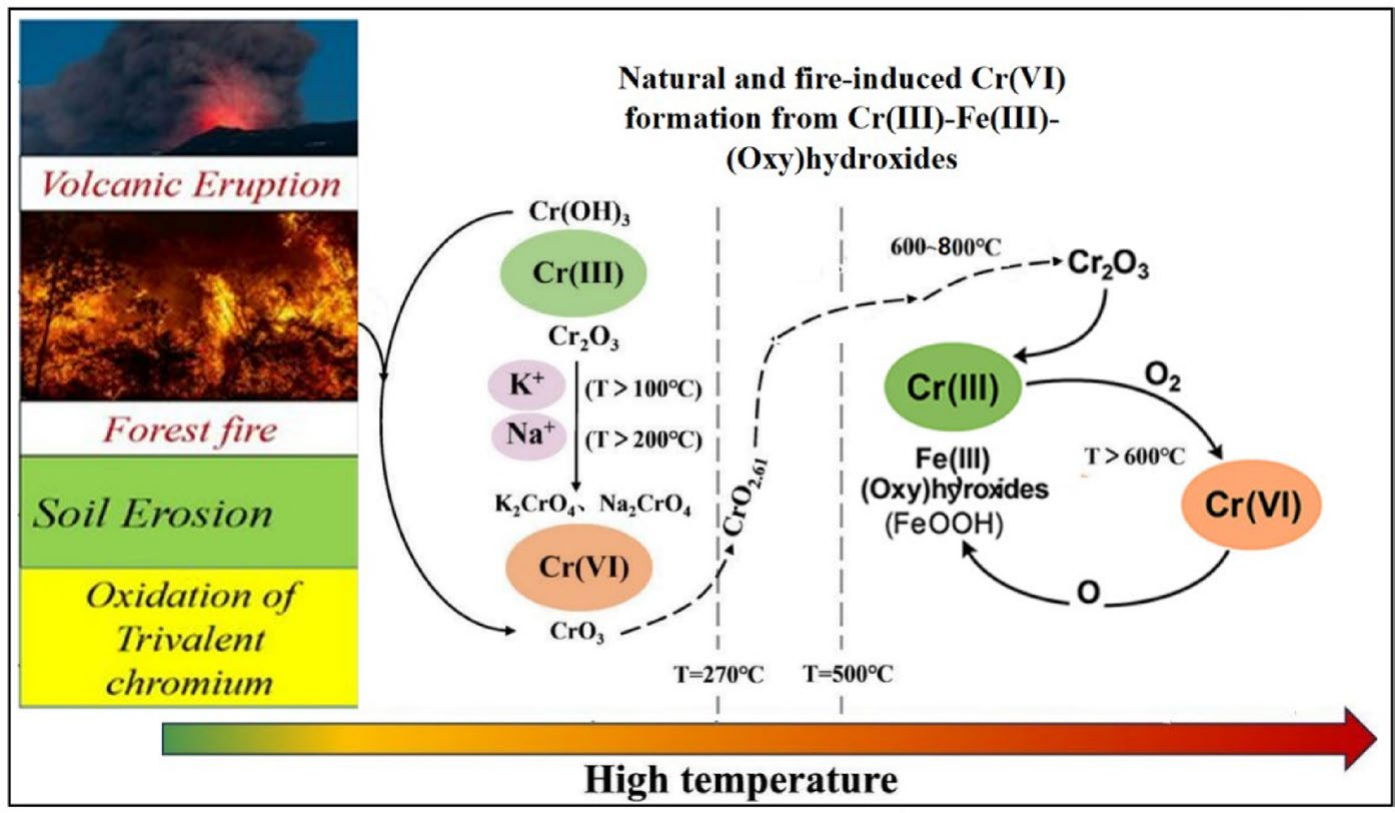
Chromium cycling in soils and groundwater, showing both natural and fire-induced Cr(VI) formation from Cr(III)-Fe(III)-(oxy)hydroxides

Wildfires can significantly alter chromium speciation by oxidizing Cr(III)-Fe(III)-(oxy)hydroxides into toxic and mobile Cr(VI) (Fig. 3), particularly in surface soils with elevated temperatures (Burton et al., 2019a, 2019b; Lázaro et al., 2022). Cr(III) is commonly hosted in iron oxides such as goethite, ferrihydrite, and hematite, either structurally incorporated or adsorbed (Luo et al., 2020). Upon heating, especially between 200–400 °C, partial dehydroxylation and phase transitions occur, promoting Cr(III) oxidation via Fe(III) electron transfer and the formation of crystalline phases like hematite and eskolaite (Cr₂O₃) (Burton et al., 2019a, 2019b).

This thermally generated Cr(VI) is often highly water-extractable and prone to leaching into groundwater, posing a significant environmental risk in fire-affected regions with shallow aquifers or steep terrain (Burton et al., 2019a, 2019b; Rascio et al., 2021; Schenk et al., 2025). Field studies confirm elevated Cr(VI) in post-fire soils, especially in areas underlain by ultramafic or serpentinite rocks (Thery et al., 2024).

Post-fire erosion and hydrologic processes can further enhance the dispersion of Cr(VI) into surrounding ecosystems (Fig. 3). As wildfires become more frequent and intense due to climate change, the release of Cr(VI) induced by fires must be considered in risk assessments and management strategies (Bolan et al., 2025; Paul et al., 2022). Understanding the mineralogical and thermal thresholds that control this process is critical for predicting contamination hotspots and guiding remediation efforts.

### Laboratory vs. environmental conditions

Cr(III) oxidation mechanisms are well-characterized in laboratory studies using strong oxidants such as δ-MnO₂, H₂O₂, or hydroxyl radicals, often under controlled pH and redox conditions (Table 3) (Liang et al., 2021; Reijonen & Hartikainen, 2016). However, in natural soils, Cr(III) is mainly found in low-solubility forms (e.g., Cr(OH)₃) or complexed with organic matter or Fe(III) oxides, limiting its reactivity. Environmental heterogeneity, reducing conditions, and uneven oxidant distribution often slow down Cr(III) transformation (Chen et al., 2024; Rahman & Thomas, 2021). Therefore, laboratory rates typically overestimate Cr(VI) formation in field settings.

**Table 3:** Comparison of Cr(III) Oxidation Pathways under Laboratory and Environmental Conditions

Aspect	Laboratory Conditions	Environmental Conditions	Key References
Dominant Oxidants	δ-MnO₂, H₂O₂, OH•	Mn oxides, H₂O₂, photochem. radicals	(Liang et al., 2021; Reijonen & Hartikainen, 2016)
	(γ-radiolysis)		
Cr(III) Forms	Cr^3^⁺, Cr(OH)^2^⁺	Cr(OH)₃, Cr–Fe/organic complexes	(Hao et al., 2022)
pH Influence	Peak oxidation near pH ~ 4	Lower reactivity at neutral to alkaline pH	(Feng et al., 2006; Qin et al., 2024)
Microbial Role	Often excluded or controlled	Affect redox; mainly reduce Cr(VI)	(Rahman & Thomas, 2021; Sharma et al., 2022)
Photochemical Paths	Active with Fe/Cr + light	Limited by light and oxidants	(Cheng et al., 2025; Djellabi et al., 2022)
Temporal Scale	Minutes to hours	Days to months	(Liang et al., 2021; Shi et al., 2022)
Oxidant Availability	Abundant, regulated	Site-specific, variable	(Chen et al., 2021; Hellerich & Nikolaidis, 2005)
System Complexity	Simplified, controlled	Complex, competing geochemical factors	(Guo et al., 2024; Kotaś & Stasicka, 2000)

### Kinetic and thermodynamic constraints

Although Cr(III) oxidation by Mn(IV) oxides is thermodynamically favorable, it is often kinetically slow in environmental systems. Cr(III) tends to form insoluble hydroxides at neutral to alkaline pH levels, which limits its interaction with oxidants. Complexation with organic ligands or Fe oxides further reduces the pool of reactive Cr^3^⁺ (Hao et al., 2022). Precipitation of Mn(II) or Cr species on Mn oxide surfaces can lead to passivation, decreasing redox reactivity (Gorny et al., 2016). Low temperatures, limited oxidant availability, and ion competition also slow down oxidation rates, which can span days to months depending on geochemistry and microbial activity (Apte et al., 2006; Varadharajan et al., 2017).

### Environmentally relevance of specific pathways

The environmental pathways of Cr(III) oxidation vary by redox conditions, mineralogy, and pH. Abiotic oxidation by δ-MnO₂ dominates in oxic soils due to its high redox potential (Feng et al., 2006; Kim et al., 2002). In anaerobic or alkaline environments, H₂O₂ can act as an oxidant, often with the assistance of Fe or Mn catalysts. Photochemical oxidation via OH• radicals contributes to the process under UV exposure, albeit with limited efficiency (Liang et al., 2021; Yen et al., 2025). Photoreduction to Cr(II), followed by oxidation to Cr(VI), adds complication. While microbial oxidation of Cr(III) is rare, microbes can indirectly influence redox conditions and Cr speciation (Kholisa et al., 2021; Ramli et al., 2023).

In summary, predicting Cr(VI) formation in natural systems requires accounting for kinetic limitations, environmental variability, and competing redox processes.

### Kinetic characteristics of Cr(III) oxidation pathways

The oxidation of Cr(III) to Cr(VI) occurs via pathways that vary significantly in rate depending on the oxidant, mineral phase, and ambient circumstances. Manganese oxides, such as birnessite (δ-MnO₂), are the most powerful oxidants under natural conditions, with apparent rate constants of 10⁻^3^–10⁻^1^ M⁻^1^ s⁻^1^ and activation energies of 40–60 kJ mol⁻^1^, indicating surface-controlled kinetics (Chen et al., 2021; Zhang et al., 2025). Surface deprotonation of Mn oxides increases reaction rates with pH, facilitating electron transfer from Cr(III) complexes (Feng et al., 2006). Oxidation by hydrogen peroxide (H₂O₂) and other reactive oxygen species is slow, with rate constants of 10⁻⁶-10⁻^5^ M⁻^1^ s⁻^1^. However, Fe(III)- or Mn(III)-catalyzed radical production can considerably increase these reactions under oxic conditions (Oze et al., 2007).

Photochemical oxidation of Cr(III) is highly dependent on ligand type and light intensity, with pseudo-first-order rate constants of 10⁻^5^–10⁻^3^ s⁻^1^ under UV or sunlight exposure (Yen et al., 2025). Microbially mediated indirect oxidation, which involves Mn- or Fe-oxidizing bacteria, typically occurs at rates one to two orders of magnitude slower than abiotic Mn-oxide reactions but can dominate in low-oxygen or circumneutral conditions due to the continuous regeneration of reactive Mn phases (Hansel et al., 2001; Wang et al., 2020). Thermal oxidation of Fe–Cr-bearing (oxyhydr)oxides during wildfires has activation energies of 75–100 kJ mol⁻^1^, resulting in fast Cr(VI) production above 300 °C and Arrhenius-type behavior (Burton et al., 2019a, 2019b; Łożyńska et al., 2025).

Overall, our kinetic results show that Cr(III) oxidation is preferred in alkaline, oxic, and high-temperature environments, but microbial-mineral coupling maintains Cr(VI) production under otherwise reducing conditions.

## Environmental behavior of Cr(VI)

The interconversion between Cr(III) and Cr(VI) in soils and sediments is driven by redox reaction influenced by pH, redox potential, organic matter, and microbial activity (Figure S1; Table S2) (Choppala et al., 2018; Kim et al., 2002). Cr(III) is oxidized to Cr(VI) in oxic and alkaline environments, mainly by Mn(IV) oxides such as birnessite (Kim et al., 2002). Conversely, Cr(VI) is reduced to Cr(III) under suboxic to anoxic conditions via reactions with Fe^2^⁺, reduced sulfur species, organic compounds, and microbial metabolites (Choppala et al., 2018). Bacteria such as *Pseudomonas*, *Shewanella*, and *Desulfovibrio* can enzymatically reduce Cr(VI), thus immobilizing it in contaminated environments (Choppala et al., 2018; Xing et al., 2025).

### Mobility, adsorption, and speciation

Cr (VI) is a mobile and toxic form of chromium derived from both natural (e.g., chromite weathering) and anthropogenic sources (e.g., electroplating, leather tanning) (Mohanty et al., 2023). In oxic, alkaline soils, Cr(VI) mainly exists as CrO₄^2^⁻ and Cr₂O₇^2^⁻ anions, which are highly soluble and weakly adsorbed (Liang et al., 2021; Sawicka et al., 2020). These species are susceptible to leaching, especially in sandy, low-organic soils. Competitive anions, such as phosphate or sulfate, can further hinder Cr(VI) sorption (Basu et al., 2014; Zhang et al., 2019).

Environmental variability, such as drought or wildfires, can destabilize immobilized Cr, reoxidizing Cr(III) back to Cr(VI) (Digiacomo et al., 2020). Thus, understanding Cr dynamics requires accounting for fluctuating redox conditions.

#### Influence of pH, TOC, clay minerals, and redox conditions

Soil pH is a key factor in Cr(VI) mobility. At low pH, positively charged Fe/Al (oxy)hydroxides (e.g., goethite, ferrihydrite) promote Cr(VI) sorption. Under alkaline conditions, the mobility of Cr(VI) increases due to electrostatic repulsion (Jiang et al., 2024; Yen et al., 2025).

Total organic carbon (TOC) can directly bind Cr(VI) through functional groups and indirectly support microbial Cr(VI) reduction (Hao et al., 2022; Rahman & Thomas, 2021). Jardine et al. (2013) quantified this relationship: (Eq. 12):

12 $$Cr\left( {VI} \right) \, adsorption \, \left( \% \right) \, = \, 704.5 \, + \, 65.7 \, \times \, TOC \, \left( \% \right) \, {-} \, 88.4 \, \times \, pH$$

Fe/Al-bearing minerals like schwertmannite, hematite, montmorillonite, and kaolinite provide reactive surfaces for Cr(VI) sorption and redox transformations (Luo et al., 2020). Fe(II)-rich minerals (e.g., biotite, green rusts) can abiotically reduce Cr(VI) to low-solubility Cr(III) hydroxides (Burton et al., 2019a).

#### Factors controlling mobility

Cr(VI) mobility is controlled by advection, sorption, redox interactions, and competition with other anions (Alidokht et al., 2021; Zulfiqar et al., 2023). Fine-textured soils may temporarily retain Cr(VI), but redox shifts, such as those occurring during groundwater table fluctuations, can trigger the reoxidation of Cr(III) by Mn oxides (Apte et al., 2006; Liang et al., 2021). These dynamics must be considered when evaluating long-term Cr fate and designing remediation strategies.

### Reduction mechanisms

The reduction of Cr(VI) to Cr(III) significantly decreases its mobility and toxicity. This process can occur via abiotic mechanisms (e.g., Fe^2^⁺, sulfides, organic matter) or through microbial enzymatic activity, depending on the redox potential, pH, and availability of electron donors (Table S3).

#### Chemical reduction (Fe(II), sulfides, organic matter)

Fe(II)-bearing minerals accelerate Cr(VI) reduction above pH 5.5, while sulfides dominate under more acidic conditions (Li et al., 2019). Reaction rates vary from seconds to hours (Gröhlich et al., 2017). Reduction produces Cr(OH)₃ and Fe/Cr hydroxides that immobilize chromium (Li et al., 2019; Unceta et al., 2010).

Key reactions:

13 $$2HCrO_{4}^{ - } \, + \, 3 \, H_{2} S \, + \, 2 \, H^{ + } \, \to \, 2 \, Cr\left( {OH} \right)_{3} \left( s \right) \, + \, 3 \, S\left( s \right) \, + \, 2 \, H_{2} O$$

14 $$Cr_{2} O_{7}^{2 - } \, + \, 6 \, Fe^{2 + } \, + \, 14 \, H^{ + } \, \to \, 2 \, Cr^{3 + } \, + \, 6 \, Fe^{3 + } \, + \, 7 \, H_{2} O$$

Humic substances and phenolic compounds can also reduce Cr(VI), but at slower rates (Zhu et al., 2023). Sulfate- and iron-reducing bacteria generate Fe^2^⁺ and H₂S, facilitating abiotic reduction in suboxic sediments (Jiang et al., 2020; Liang et al., 2021).

#### Microbial reduction

**Fig. 4:**
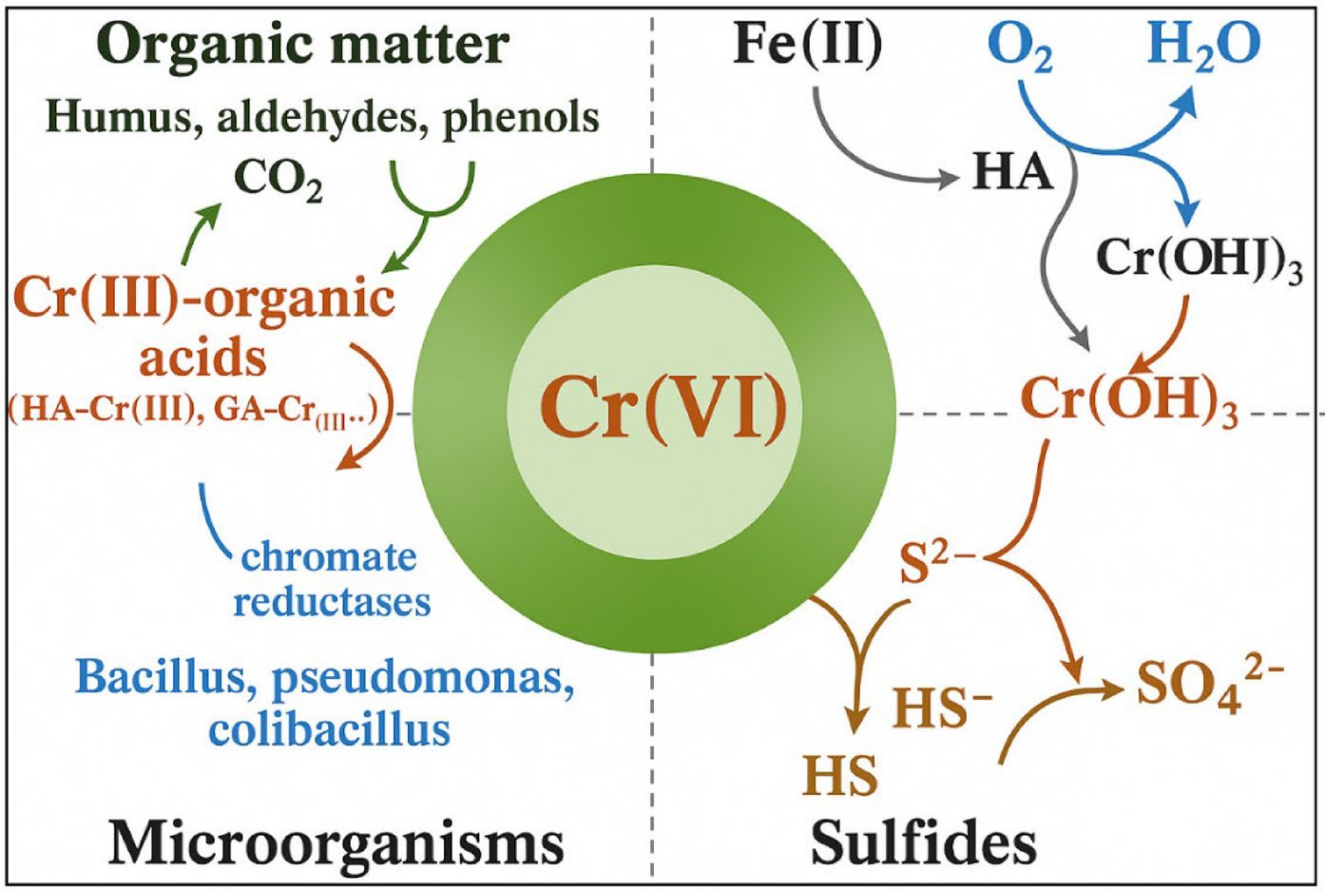
The reduction of Cr(VI) through chemical and microbial paths (Liang et al., 2021)

Microorganisms reduce Cr(VI) (see Fig. 4), either directly via chromate reductases (e.g., ChrR) or indirectly by generating Fe^2^⁺ or S^2^⁻ (Huang et al., 2023; Pang et al., 2024; Ramli et al., 2023). Anaerobic conditions enhance microbial reduction, where Cr(VI) acts as a terminal electron acceptor (Pang et al., 2024). Electron donors, such as lactate, formate, and pyruvate, are essential for this process (Rahman & Thomas, 2021).

Microbial reduction can be inhibited by high Cr(VI) concentrations or limited donor availability (Sharma et al., 2022; Wu et al., 2022). Still, indigenous bacteria (e.g., *Shewanella*, *Geobacter*, *Pseudomonas*) can reduce over 99% of dissolved Cr(VI) within 96 h under anaerobic conditions (Ren et al., 2023; Su et al., 2023).

Key reaction:

15 $$CrO_{4}^{2 - } \, + \, organic \, carbon \, \left( {e.g., \, lactate} \right) \, \to \, Cr\left( {OH} \right)_{3} \, \left( s \right) \, + \, CO_{2}$$

##### Microbial indirect regulation of Cr(III) oxidation

Microorganisms have an important indirect role in Cr(III) oxidation by facilitating the regeneration of redox-active minerals and creating reactive intermediates that support abiotic oxidation cycles. Mn(II)-oxidizing bacteria, such as *Leptothrix discophora* and *Pseudomonas putida*, can catalyze the oxidation of Mn(II) to Mn(III, IV) oxides in oxic or suboxic environments (Tebo et al., 2005; Wang et al., 2020). These newly generated Mn oxides have a high oxidative potential and can subsequently convert Cr(III) to Cr(VI), combining microbial metabolism with abiotic Cr transformation (Hansel et al., 2001). Similarly, Fe(II)-oxidizing bacteria (e.g., *Gallionella ferruginea, Leptothrix ochracea*) assist in producing Fe(III)-(oxyhydr)oxides, which act as catalytic surfaces for Mn regeneration and Cr oxidation (Tang et al., 2014). Aside from mineral regeneration, microbial communities create reactive metabolites such as superoxide and hydrogen peroxide, which promote indirect Cr(III) oxidation via redox chain reactions (Diaz et al., 2013). These findings show a bio-abiotic coupling mechanism in which microbial activity supports oxidative mineral phases that drive Cr(VI) persistence and cycling in soil-groundwater systems. Understanding this association is essential for forecasting Cr dynamics under varying redox and microbial environments.

#### Influencing factors

Acidic pH enhances Cr(VI) reduction by promoting stronger interactions between reductants and chromate species (Shao et al., 2025). Low redox potential and sufficient electron donors further promote reduction (Liang et al., 2021). Mn-doped ferrihydrite enhances Cr(VI) immobilization by increasing redox buffering capacity (Liang et al., 2021).

#### Reduction products

Reduction of Cr(VI) leads to Cr(OH)_3_ and Fe–Cr hydroxide precipitates that are stable under reducing conditions (Li et al., 2022). These solid phases limit Cr mobility and contribute to long-term stabilization of contaminated soils and aquifers (Beretta et al., 2024; Franco et al., 2009).

## Risk assessment and environmental impacts of Cr(VI)

### Ecotoxicity and microbial disruption

**Fig. 5:**
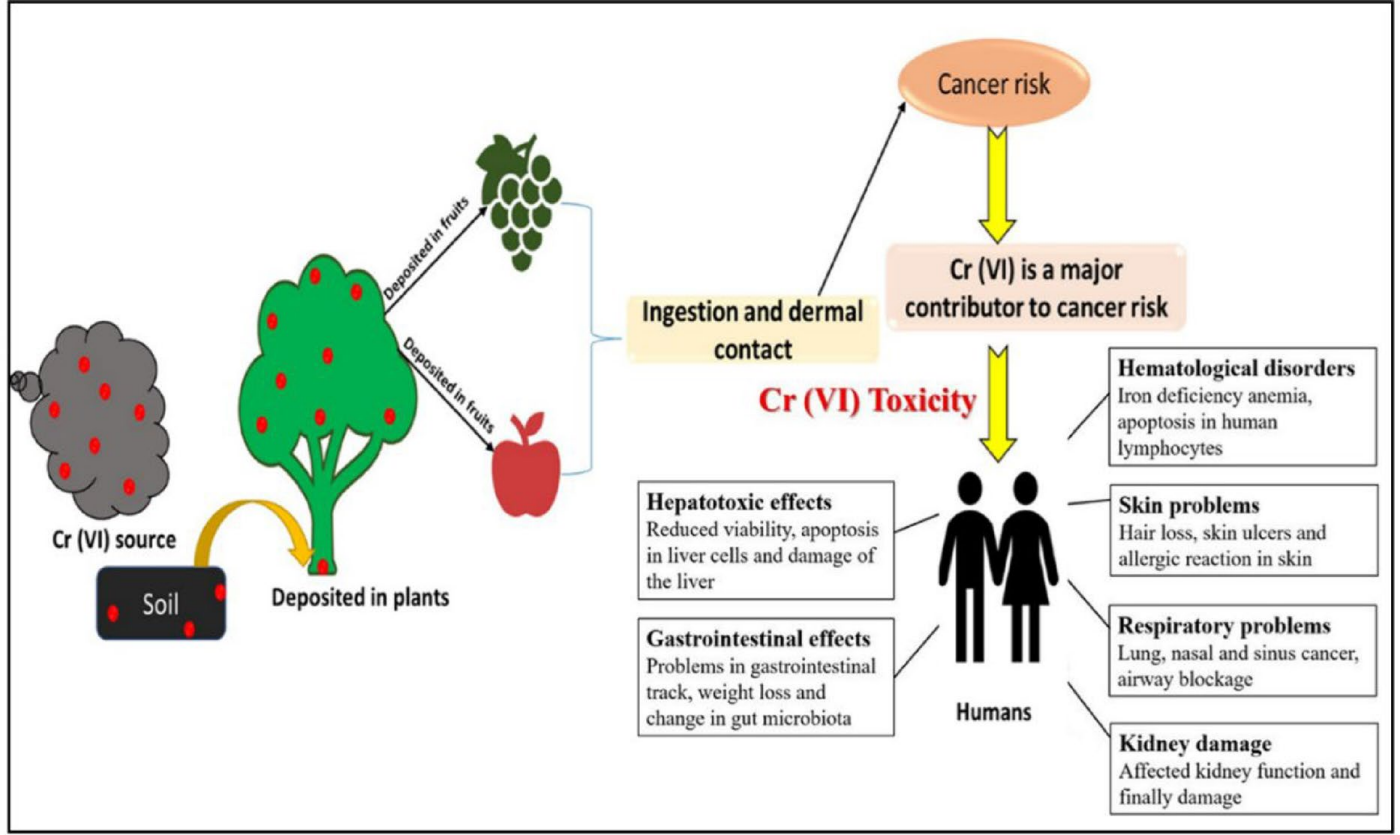
Hexavalent chromium (Cr(VI)) has effects on both the environment and human health

Cr(VI) is a highly mobile and bioavailable toxicant that enters microbial cells via anion channels (e.g., sulfate transporters), where it is reduced to Cr(III), generating reactive intermediates (Cr(V), Cr(IV)) and reactive oxygen species (ROS**)** (Fig. 5) (Sharma et al., 2022). This induces oxidative stress, DNA damage, and protein/lipid degradation, disrupting microbial functions such as nitrogen fixation and enzymatic activity (Prasad et al., 2021).

Such disruptions impair nutrient cycling, organic matter decomposition, and microbial diversity. Sensitive organisms like arbuscular mycorrhizal fungi and rhizobacteria are especially vulnerable, undermining plant symbioses and soil fertility (Chen et al., 2021; Nikolaou et al., 2022). In aquatic systems, Cr(VI) concentrations as low as 0.005–0.01 mg L⁻^1^ impair microbial metabolism and reduce biodiversity, destabilizing food webs (Tu et al., 2025).

### Impact on soil functions and biodiversity

Cr(VI) negatively affects soil enzymatic processes (e.g., urease, dehydrogenase), microbial respiration, and nutrient cycling (Prasad et al., 2021). Its persistence in sandy, low-organic soil increases environmental risk. Phytotoxic effects include inhibited seed germination, reduced growth, chlorosis, and impaired nutrient uptake. At the cellular level, Cr(VI) disrupts chloroplasts and photosynthesis, resulting in reduced biomass and yield (Sharma et al., 2022). In hydroponic systems, 0.5 mg L⁻^1^ can significantly inhibit photosynthetic performance (Nikolaou et al., 2022). Cr(VI) bioaccumulates in food chains (Fig. 5), affecting higher organisms such as pollinators and birds, and contributing to biodiversity loss and reduced ecosystem resilience (Angon et al., 2024).

### Human health and water contamination risks

Cr(VI) is a known human carcinogen (IARC Group 1), associated with gastrointestinal and respiratory cancers, nephrotoxicity, hepatotoxicity, anemia, and immune suppression (Georgaki & Charalambous, 2022; Sharma et al., 2022). Health risks arise from ingestion, inhalation, and dermal exposure (Fig. 5). Vulnerable populations include children, older adults, and communities that rely on shallow groundwater (Den Braver-Sewradj et al., 2021).

Elevated Cr(VI) contamination in soils and groundwater has been widely recorded in both industrial and natural environments worldwide. Groundwater in Kanpur, India, a major leather-tanning and electroplating hub, contains Cr(VI) concentrations ranging from 0.05 to 16.3 mg L⁻^1^, far exceeding the WHO and U.S. EPA drinking-water limits of 0.05 and 0.1 mg L⁻^1^, respectively (Singh et al., 2019; Prasad et al., 2021). The contamination is mostly caused by untreated tannery effluents and solid waste leaching, with health risk evaluations indicating substantial carcinogenic and non-carcinogenic risks from drinking water and skin exposure.

In Hinkley, California (USA), a well-documented incidence of industrial groundwater contamination from cooling-tower discharges by Pacific Gas and Electric resulted in Cr(VI) values up to 580 µg L⁻^1^, affecting an area of more than 10 km^2^ (California Water Board, 2020; Izbicki & Groover, 2023). Long-term exposure has been linked to increased cancer risk, gastrointestinal issues, and oxidative DNA damage, necessitating significant cleanup under the US EPA Superfund program. In Yunnan Province, China, chromite mining has resulted in groundwater Cr(VI) levels of 0.1–2.8 mg L⁻^1^ (Zhao et al., 2020), while in the Asopos Basin, Greece, industrial discharge and ophiolitic weathering produce concentrations of 0.03–1.6 mg L⁻^1^, posing significant carcinogenic risks (Kelepertzis et al., 2015).

Chromite-affected aquifers in Northern Pakistan have Cr(VI) values ranging from 0.08 to 3.9 mg L⁻^1^, primarily due to chromite ore processing and tannery waste, posing nephrotoxicity and genotoxicity hazards (Rashid et al., 2023; Younas et al., 2023). Cr(VI) accumulation in aquatic biota can cause oxidative stress, endocrine disturbance, and reproductive failure at levels as low as 0.01 mg L⁻^1^. These global cases show that Cr(VI) pollution is caused by both industrial and geogenic processes, such as tannery effluent discharge, chromite mining, and the oxidation of Cr(III)-bearing rocks, and that its persistence in aquifers poses long-term ecological and human health problems. To reduce Cr(VI) exposure and protect groundwater quality, effective risk management needs regular monitoring, enforcement of environmental regulations, and region-specific remediation solutions.

## Chromium remediation strategies

Biological treatments are best suited for low to moderate Cr(VI) contamination or as polishing steps after primary remediation. When integrated into comprehensive site plans, they enhance sustainability and restoration outcomes (Xing et al., 2025).

### Physical and chemical approaches

Effective remediation often requires integrating physical, chemical, and biological methods (Figure S2, Table 4), tailored to site-specific conditions (Acharyya et al., 2023). Among novel materials, engineered biochar, such as CTAB-modified peanut shell biochar, can achieve 97–100% Cr(VI) immobilization via enhanced electrostatic attraction and surface functionalization (Murad et al., 2022). Traditional methods remain essential, particularly at highly contaminated sites. Pump-and-treat systems are reliable but energy-intensive and slow (Bortone et al., 2020).

**Table 4:** Summary of chromium remediation strategies, their advantages, and disadvantages

Remediation Method	Category	Mechanism	Advantages	Disadvantages	References
Engineered Biochar	Physical/Chemical	Cr(VI) adsorption via surface functionalization	Low-cost, eco-friendly, dual-use, efficient	Needs modification; less effective at high loads	(Liang et al., 2021; Murad et al., 2022)
Pump-and-Treat	Physical	Groundwater extraction and off-site treatment	Established, controllable, and handles large plumes	Expensive, energy-intensive, long remediation time	(Bortone et al., 2020)
Permeable Reactive Barriers	Physical/Chemical	In situ Cr(VI) reduction via reactive media (ZVI)	Passive, low upkeep, reduces mobility	Clogging risk, hydrogeology limits, Cr(III) remobilization	(Fang et al., 2021; Xing et al., 2025)
Soil Washing	Chemical	Cr(VI) desorption using acids/chelators	High removal in hotspots, ex-situ application	Generates liquid waste; less effective in clays	(Xing et al., 2025)
Solidification/Stabilization	Chemical	Encapsulation or Cr(VI) to Cr(III) conversion	Stable, lowers leachability, construction use	Alters soil; may degrade over time	(Komaei et al., 2025)
Redox Manipulation	Chemical	Injecting reducers to form Cr(III)	In situ, boosts natural attenuation	Requires careful control; may harm microbiota	(Tumolo et al., 2022)
Bioremediation	Biological	Microbial Cr(VI) reduction	Eco-friendly, improves soil, in situ viable	pH/nutrient sensitive; slow	(Ramli et al., 2023; Zhang et al., 2025)
Phytoremediation	Biological	Plant uptake/sequestration of Cr	Cheap, improves ecology, low-tech	Shallow roots, slow, biomass disposal needed	(Aryal, 2024; Xing et al., 2025)
Biosorption	Biological	Cr(VI) binds to biomass surfaces	Low-cost, reusable, suitable for dilute solutions	Not for in situ soil; regeneration/disposal issues	(Mushtaq et al., 2022)
Monitored Natural Attenuation	Integrated	Relies on natural dilution, sorption, and reduction	Low-cost, minimal disruption	Long monitoring; ineffective for high concentrations	(Hellerich & Nikolaidis, 2005; Xing et al., 2025)

Permeable reactive barriers (PRBs) with zero-valent iron (ZVI) offer passive in situ treatment but may lose reactivity over time (Fang et al., 2021; Xing et al., 2025). Soil washing uses acids or chelators to extract Cr(VI) into a treatable liquid phase but generates secondary waste (Xing et al., 2025).

Solidification/stabilization (S/S) combines soil with binders (e.g., cement or lime) to reduce Cr(VI) leachability, though it alters soil structure (Komaei et al., 2025; Shen et al., 2018). Redox manipulation—injecting Fe^2^⁺, sulfides, or organics—promotes in situ Cr(VI) reduction.

Surface water remediation is gaining attention, especially in post-fire landscapes where erosion enhances Cr(VI) runoff. Vegetative stabilization, iron-based sorbents, and biochar amendments can reduce Cr(VI) mobility and safeguard downstream water bodies (Ji et al., 2022).

### Biological approaches

Bioremediation uses Cr(VI)-reducing microbes such as *Pseudomonas* and *Bacillus*, often enhanced by electron donors like acetate or molasses (Xing et al., 2025; Zhang et al., 2025). Phytoremediation with plants like *Sedum plumbizincicola* reduces Cr bioavailability via uptake, sequestration, and root-driven microbial enhancement (Aryal, 2024; Xing et al., 2025).

Biosorption employs algae, fungi, or biofilms to passively remove Cr(VI) from water, suitable for industrial effluents due to low cost and high efficiency (Mushtaq et al., 2022). Combined plant–microbe systems offer synergistic remediation benefits.

Advantages of biological techniques include minimal energy demand and enhanced soil health. However, limitations such as slow kinetics, environmental sensitivity, and biomass disposal must be addressed. Phytoremediation is limited by rooting depth and seasonal growth, while biosorption requires scalable systems and biosorbent management (Aryal, 2024).

### Natural attenuation and redox manipulation

Monitored Natural Attenuation (MNA) passively reduces Cr(VI) via naturally occurring Fe^2^⁺, sulfides, and organic matter, forming stable Cr(OH)₃ or Fe–Cr precipitates (Zhao et al., 2017). Even in oxic zones, Fe^2^⁺ can support reduction (Burton et al., 2019a, 2019b).

Enhancing MNA redox manipulation involves amendments such as molasses, PHB, or yeast extract. PHB supports slow-release carbon delivery; yeast extract can achieve complete Cr(VI) reduction within 7 days (Tumolo et al., 2022; Xing et al., 2025).

Successful implementation requires long-term monitoring of redox potential, pH, dissolved oxygen, and chromium speciation. Monitoring networks must detect possible Cr(III) reoxidation via Mn oxides (Apte et al., 2006; Xie et al., 2025).

Cr(III) reoxidation remains a major limitation in long-term remediation of chromium-contaminated soils and aquifers. The phenomenon is commonly triggered by multiple concurrent mechanisms, including (i) alkaline pH and high oxygen availability, which enhance the redox potential of the system and promote Cr(III) oxidation on mineral surfaces (Oze et al., 2007; Tu et al., 2025); (ii) regeneration of Mn oxides from Mn(II) under oxic or semi-oxic conditions, which restores the oxidizing capacity of Mn-bearing minerals (Tang et al., 2014; Yan et al., 2022); and (iii) indirect microbe-mediated oxidation**,** where Mn- or Fe-oxidizing bacteria catalyze redox cycling and accelerate Cr(VI) regeneration (Wang et al., 2020).

The relative contribution of these methods is determined by environmental characteristics such as pH, oxygen diffusion, organic matter content, and mineral aging (Liang et al., 2021; Zhang et al., 2025). To mitigate the rebound effect, anti-reoxidation optimization measures should be incorporated into remediation design. Maintaining slightly reducing conditions in chemical or mineral-based systems via Fe(II)-bearing phases or organic additions might inhibit Mn oxide regeneration and stable Cr as Cr(III) (Jeong & Claus, 2013).

Controlling redox gradients and maintaining persistent anaerobic microzones during microbial or biochar-assisted remediation can help to minimize oxidative rebounds (Chen et al., 2024; Gao et al., 2020). Furthermore, frequent in situ monitoring of redox potential, Mn speciation, and dissolved oxygen can aid in detecting the onset of reoxidation and guiding adaptive management (Burton et al., 2019a, 2019b; Varadharajan et al., 2017). Incorporating these targeted controls can improve the durability and reliability of Cr(VI) immobilization in dynamic soil-groundwater systems.

Molecular tools (e.g., eDNA) aid in assessing microbial communities, while multivariate analysis (e.g., PCA, MANOVA) clarifies biogeochemical controls and informs adaptive remediation strategies.

## Conclusions and future research directions

This review highlights the complex geochemical, microbial, photochemical, and thermal mechanisms that drive the oxidation of Cr(III) to the toxic Cr(VI) in soil and groundwater. Oxidants like MnO₂ and H₂O₂, along with photolysis and wildfire-induced heating, contribute to Cr(VI) generation, particularly under alkaline, oxic, and post-fire conditions. These pathways are influenced by pH, redox potential, mineralogy, and the presence of organic matter. Despite reduced industrial emissions, Cr(VI) remains a persistent threat due to its solubility, mobility, and potential for re-oxidation.

Bio-reduction, biosorption, and reactive minerals (e.g., Mn-doped ferrihydrite) offer promising remediation strategies, but their long-term effectiveness is often limited by environmental variability and mineral aging. This review provides a comprehensive synthesis of the mechanistic pathways governing Cr(III) oxidation to Cr(VI) in soil-groundwater systems, highlighting both classical and emerging processes. It integrates recent advances on thermally induced and microbially mediated oxidation, along with the roles of mineralogical transformations and environmental conditions in controlling Cr redox dynamics. By linking mechanistic insights to environmental implications and remediation potential, this work establishes a framework for predicting Cr(VI) behavior in complex natural systems. The insights offered herein are intended to influence future experimental and field studies targeted at reducing chromium toxicity and enhancing long-term management of Cr-contaminated ecosystems. Future research should quantify Cr(VI) formation under fire conditions, refine detection methods (e.g., isotopic tracing), and explore microbial diversity for robust bioremediation. Field trials with low-cost materials (e.g., iron oxides, schwertmannite, biochar) in diverse soils are needed to optimize immobilization. Integrating redox chemistry, hydrology, and microbiology into predictive models is essential for adaptive management. Emphasis should be placed on green, cost-effective solutions that both remove Cr(VI) and minimize re-oxidation risks.

From a management perspective, incorporating Cr(VI) risk into post-fire land restoration strategies and groundwater protection plans is increasingly necessary. Policymakers should prioritize surveillance in vulnerable areas, including wildfire-prone regions, former industrial sites, and shallow aquifers. Furthermore, regulatory frameworks must reflect the dynamic nature of chromium speciation, promoting adaptive remediation that anticipates re-oxidation risks and supports long-term soil and water quality protection.

## Supplementary Information

Below is the link to the electronic supplementary material.

Supplementary file1 (DOCX 1481 kb)

## Data Availability

The review article data are based on previously published research. All data cited in the literature are publicly available.
