# Supplementary material for: Mechanistic insights and environmental ramifications of Cr(III) oxidation to Cr(VI) in soil and groundwater systems: bridging geochemical mechanisms and emerging remediation strategies
Source: Environ Geochem Health. 2025 Nov 24;48(1):12. doi: 10.1007/s10653-025-02901-2 (PMC12644231; doi:10.1007/s10653-025-02901-2)
Supplement: Supplementary file 1 — Supplementary file1 (DOCX 1481 kb) [file 10653_2025_2901_MOESM1_ESM.docx]

**Mechanistic Insights and Environmental Ramifications of Cr(III) Oxidation to Cr(VI) in Soil and Groundwater Systems: Bridging Geochemical Mechanisms and Emerging Remediation Strategies**

Atta Rasool^1,*^, Eva Pertile^1^, Kateřina Brožová^1^, Jan Halfar^1^, Kristina Čabanová^1^, Petra Malíková^1^, Jitka Chromíková^1^, Oldřich Motyka^1^, Silvie Drabinová^1^, Silvie Heviánková^1^

^1^Faculty of Mining and Geology, VSB–Technical University of Ostrava, 17. listopadu 2172/15, Ostrava-Poruba, 708 00, Czech Republic


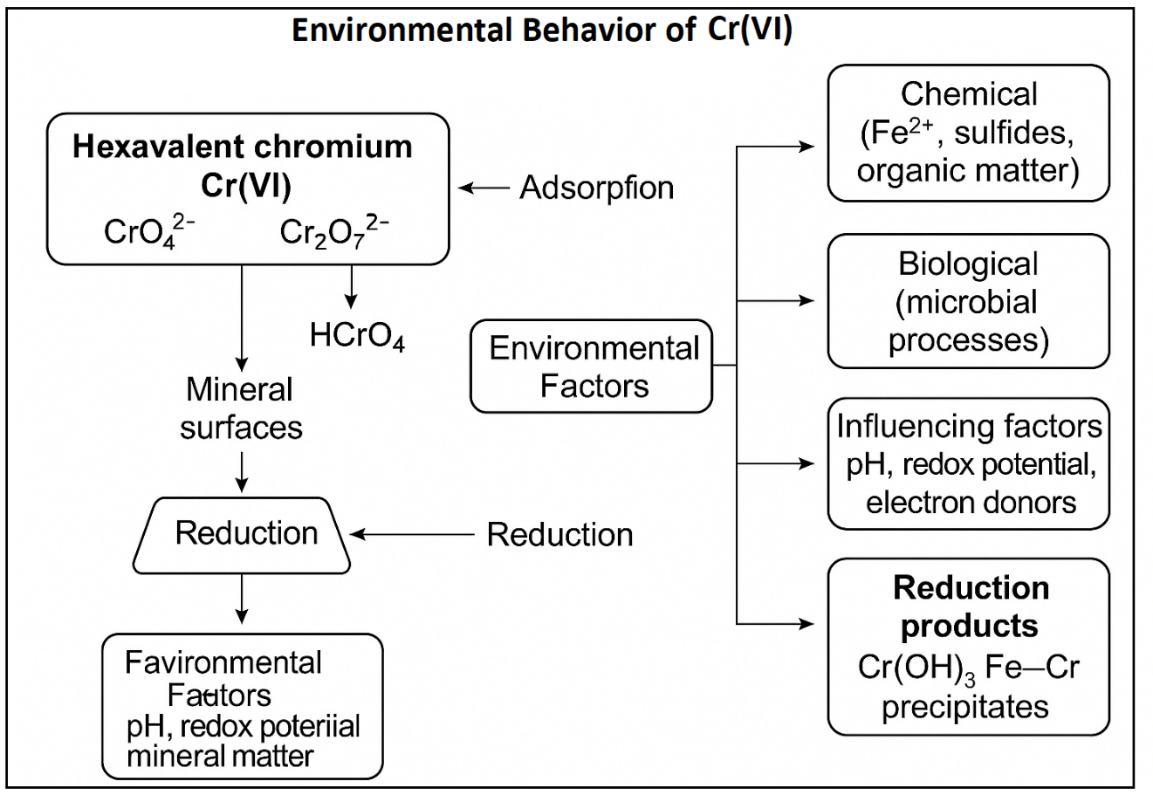


**Figure S1.** Cr(VI) mobility and reduction pathways. Adsorption, redox conditions, and environmental factors control


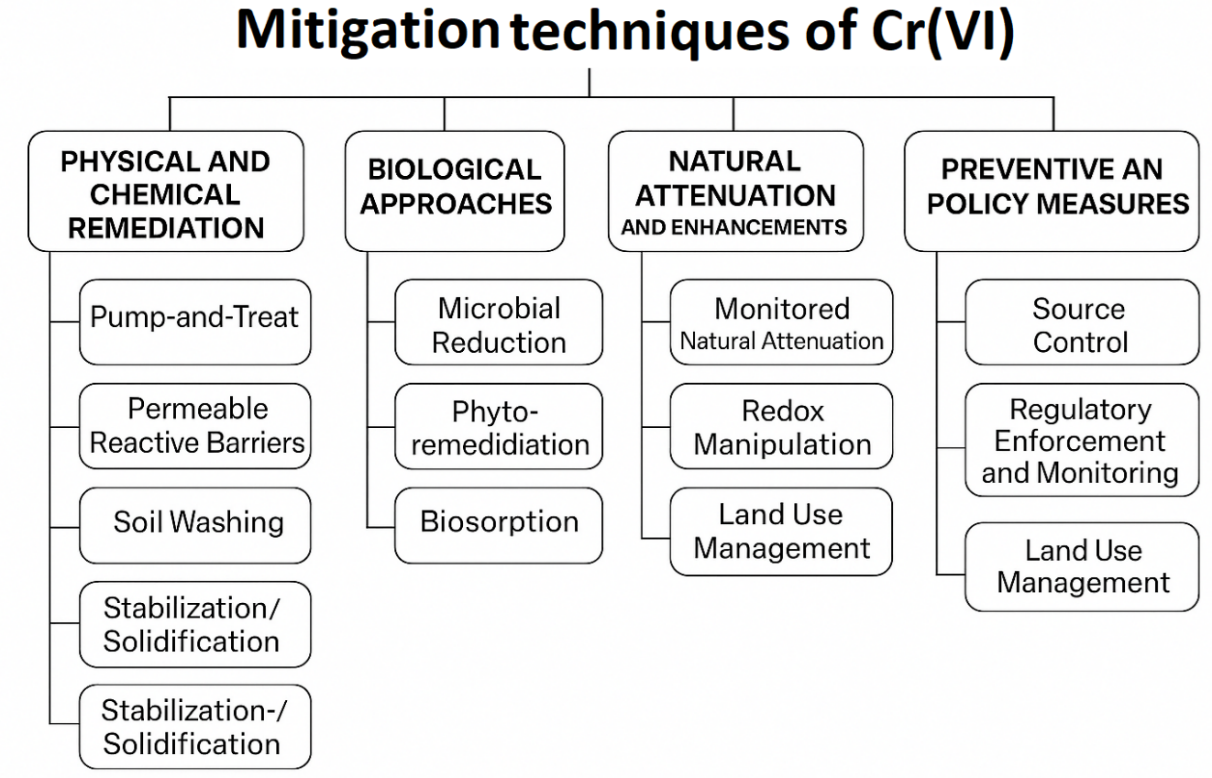


**Figure S2.** Mitigation Strategies of Cr(VI) Contamination using different approaches, i.e., physical, chemical, and biological.

**Table S1.** Cr(III) and Cr(VI) Concentrations in Groundwater and Surface Waters

| **Region** | **Water Type** | **Cr(VI) (µg/L)** | **Cr(III) (µg/L)** | **Key Characteristics** | **Reference(s)** |
| --- | --- | --- | --- | --- | --- |
| Antelope Valley, CA | Groundwater (natural) | <1–32 | Typically <10% of total Cr | Alkaline (pH ~8), oxic, long residence, Cr-bearing schist | (Izbicki et al., 2015) |
| West-side Central Valley, CA | Groundwater (agricultural) | <1–69 | Low | Cr-rich alluvium, irrigation return, high nitrate, alkaline oxic aquifer | (Guo et al., 2020) |
| Loess Plateau, China | Groundwater | <4–300 | Not quantified | Loess fissure‑pore aquifers; pH 7.8–8.8; high Eh; amphibole/tremolite | (Ning et al., 2025) |
| Jingyang County, China | Groundwater | Up to 300 | Not quantified | Loess tableland, weathered sandstone, slow groundwater flow | (Ning et al., 2025) |
| Troodos and coastal aquifers, Cyprus | Groundwater/Spring | ND–26 | ND-few (indirect) µg/L | Ultramafic/mafic ophiolite; fractured aquifers; meteoric recharge | (Zissimos et al., 2021) |
| Aosta industrial area, Italy | Groundwater (industrial) | 0.09–165 | Not reported | Superficial steel slag deposits, Ca–Mg–HCO₃/SO₄ basins | (Grappein et al., 2021) |
| Tuscany/Cecina coast, Italy | Groundwater (natural/coastal) | Up to 73 | Not reported | Ophiolitic rocks, Mn-oxide oxidation in coastal alluvium | (Bastianoni et al., 2021) |
| Aegean and Central Greece | Groundwater/tap water (urban) | Up to 156 | Not reported | Ophiolitic formations + industrial (fly-ash/waste); some public supply exceeding EU limit | (Drougas et al., 2025) |
| Loess Plateau, China | Surface water | 12-May | Not quantified | Seasonal recharge, moderate DO/Eh | (Ning et al., 2025) |
| Bauru Basin, São Paulo, Brazil | Groundwater | Up to 130 | Not reported | Sandstone aquifer; high pH >10; Mn-oxide–catalyzed oxidation of Cr(III); chrome-diopside source | (Bertolo et al., 2011) |
| New Caledonia (ultramafic soils) | Soil pore-water | Up to 700 | Not reported | Phosphate-amended serpentine soil; Mn-mediated Cr(III) oxidation | (Becquer et al., 2003) |
| León Valley, Mexico | Groundwater | ~12 | Not reported | Ultramafic geology (Sierra Guanajuato); geogenic Cr(VI) source | (Villalobos-Aragón et al., 2012) |
| Chromite mining area | Surface water | 20-500 | 30-May | Ultramafic rock weathering; mine drainage; oxidizing to slightly reducing | (Kokkinos et al., 2024; Rashid et al., 2023) |
| Cyprus (Kiti–Pervolia) | GW | Median 4.5 | Not reported | Coastal agricultural recharge | (Zissimos et al., 2021) |
| Greece (ultramafic areas) | Tap/GW (urban) | >2 up to 156 | Not reported | Ultramafic geology, Mn-catalyzed oxidation, EU exceedances | (Dermatas et al., 2015) |
| Southern India (tannery areas) | GW | 10 - 190 (0.01-0.19 mg/L) | 0.01- 0.19 mg/L total Cr | Industrial tanning contamination exceeding WHO limit (0.05 mg/L) | (Ali et al., 2022; Brindha and Elango, 2012) |

**Table S2.** Environmental behavior, migration, transformation and fate of Cr(VI)

| **Process** | **Description** | **Environmental Impact** | **Reference** |
| --- | --- | --- | --- |
| Leaching and Migration | Cr(VI) moves through unsaturated and saturated zones | Groundwater contamination risk | (Wang et al., 2020; Wei et al., 2021) |
| Adsorption/Desorption | Cr(VI) weakly adsorbs to oxides and clays; desorption under alkaline pH | Enhanced mobility under high pH | (Liang et al., 2021) |
| Microbial Reduction | Reduction to Cr(III) via Fe(III)- or sulfate-reducing bacteria | Localized immobilization | (Huang et al., 2024) |
| Abiotic Reduction | Reduction by Fe²⁺, sulfides, and organic matter | Cr(VI) sequestration in anoxic zones | (Hou et al., 2023) |
| Post-Fire Redistribution | Cr(VI) formed during fires is water soluble and rapidly mobilized by rain | Rapid surface and subsurface transport | (Burton et al., 2019) |

**Table S3.** Overview of Common Cr(VI) Reducing Agents

| **Reducing Agent** | **Formula** | **Approx. Redox Potential** | **Environmental Relevance** | **Limitations Reference** | |
| --- | --- | --- | --- | --- | --- |
| Ferrous iron | Fe²⁺ | +0.77 V (vs. SHE) | Naturally present in suboxic soils and sediments | Ineffective in oxic conditions; pH-sensitive | (Liang et al., 2021) |
| Zero-valent iron | Fe⁰ | –0.44 V | Widely used in permeable reactive barriers (PRBs) and soil treatments | Surface passivation; limited penetration in compact soils | (Liang et al., 2021; Verma et al., 2025) |
| Sulfides | HS⁻, S²⁻ | ~–0.24 to –0.27 V | Produced via microbial sulfate reduction in anoxic environments | H₂S toxicity; redox sensitive | (Choppala et al., 2018; Kushkevych et al., 2019) |
| Organic matter | – | Variable (–0.2 to –0.5 V) | Abundant in soils; supports microbial and abiotic Cr(VI) reduction | Variable efficiency; influenced by bioavailability and structure | (Choppala et al., 2018; Ren et al., 2023) |
| Calcium polysulfide | CaSₓ | Not defined | Applied in field-scale remediation of Cr(VI)-contaminated sites | Secondary byproducts (e.g., SO₄²⁻); handling precautions | (Graham et al., 2006; Moon et al., 2008) |
| Sodium dithionite | Na₂S₂O₄ | ~–0.66 V | Rapid-acting reductant in soil washing and ex situ treatments | Instability; short lifetime in solution | (Fan et al., 2023; Telfeyan et al., 2019) |

Note: Redox potentials are standard vs SHE and may vary with pH and matrix.

**Reference**

Ali, H.Q., Yasir, M.U., Farooq, A., Khan, M., Salman, M., & Waqar, M. (2022). Tanneries impact on groundwater quality: A case study of Kasur city in Pakistan. *Environmental Monitoring and Assessment, 194*, 823. [https://doi.org/10.1007/s10661-022-10502-0](https://doi.org/10.1007/s10661-022-10502-0?utm_source=chatgpt.com)

Bastianoni, A., Guastaldi, E., Barbagli, A., Bernardinetti, S., Zirulia, A., Brancale, M., & Colonna, T. (2021). Multivariate analysis applied to aquifer hydrogeochemical evaluation: A case study in the coastal significant subterranean water body between “Cecina River and San Vincenzo”, Tuscany (Italy). *Applied Sciences, 11*, 7595. [https://doi.org/10.3390/app11167595](https://doi.org/10.3390/app11167595?utm_source=chatgpt.com)

Bertolo, R., Bourotte, C., Hirata, R., Marcolan, L., & Sracek, O. (2011). Geochemistry of natural chromium occurrence in a sandstone aquifer in Bauru Basin, São Paulo State, Brazil. *Applied Geochemistry, 26*, 1353–1363. [https://doi.org/10.1016/j.apgeochem.2011.05.009](https://doi.org/10.1016/j.apgeochem.2011.05.009?utm_source=chatgpt.com)

Brindha, K., & Elango, L. (2012). Impact of tanning industries on groundwater quality near a metropolitan city in India. *Water Resources Management, 26*, 1747–1761. [https://doi.org/10.1007/s11269-012-9985-4](https://doi.org/10.1007/s11269-012-9985-4?utm_source=chatgpt.com)

Dermatas, D., Mpouras, T., Chrysochoou, M., Panagiotakis, I., Vatseris, C., Linardos, N., Theologou, E., Boboti, N., Xenidis, A., Papassiopi, N., & Sakellariou, L. (2015). Origin and concentration profile of chromium in a Greek aquifer. *Journal of Hazardous Materials, 281*, 35–46. [https://doi.org/10.1016/j.jhazmat.2014.09.050](https://doi.org/10.1016/j.jhazmat.2014.09.050?utm_source=chatgpt.com)

Drougas, C., Kelepertzis, E., Kypritidou, Z., Sigala, E., Matiatos, I., Dotsika, E., Vasileiou, E., Louloudis, G., Mertiri, E., Boeckx, P., Oikonomopoulos, E., & Roumpos, C. (2025). Controls on the geochemical composition of surface water in Alfeios River basin in the transition era of lignite mine closure at Megalopolis, Greece. *Science of the Total Environment, 970*, 179006. [https://doi.org/10.1016/j.scitotenv.2025.179006](https://doi.org/10.1016/j.scitotenv.2025.179006?utm_source=chatgpt.com)

Fan, J., Liu, C., Zheng, J., & Song, Y. (2023). Dithionite promoted microbial dechlorination of hexachlorobenzene while goethite further accelerated abiotic degradation by sulfidation in paddy soil. *Ecotoxicology and Environmental Safety, 259*, 115047. [https://doi.org/10.1016/j.ecoenv.2023.115047](https://doi.org/10.1016/j.ecoenv.2023.115047?utm_source=chatgpt.com)

Graham, M.C., Farmer, J.G., Anderson, P., Paterson, E., Hillier, S., Lumsdon, D.G., & Bewley, R.J.F. (2006). Calcium polysulfide remediation of hexavalent chromium contamination from chromite ore processing residue. *Science of the Total Environment, 364*, 32–44. [https://doi.org/10.1016/j.scitotenv.2005.11.007](https://doi.org/10.1016/j.scitotenv.2005.11.007?utm_source=chatgpt.com)

Grappein, B., Lasagna, M., Capodaglio, P., Caselle, C., & Luca, D.A.D. (2021). Hydrochemical and isotopic applications in the western Aosta Valley (Italy) for sustainable groundwater management. *Sustainability, 13*, 487. [https://doi.org/10.3390/su13020487](https://doi.org/10.3390/su13020487?utm_source=chatgpt.com)

Guo, H., Chen, Y., Hu, H., Zhao, K., Li, H., Yan, S., Xiu, W., Coyte, R.M., & Vengosh, A. (2020). High hexavalent chromium concentration in groundwater from a deep aquifer in the Baiyangdian Basin of the North China Plain. *Environmental Science & Technology, 54*, 10068–10077. [https://doi.org/10.1021/acs.est.0c02357](https://doi.org/10.1021/acs.est.0c02357?utm_source=chatgpt.com)

Hou, J., Li, Z., Xia, J., Miao, L., Wu, J., & Lv, B. (2023). Role of sulfate-reducing bacteria in the removal of hexavalent chromium by biosynthetic iron sulfides (FeS1+x). *Water, 15*, 1589. [https://doi.org/10.3390/w15081589](https://doi.org/10.3390/w15081589?utm_source=chatgpt.com)

Huang, H., Gao, Y.-J., Cao, Z.-X., Tian, Z.-Q., Bai, Y.-F., Tang, Z.-X., Ali, A., Zhao, F.-J., & Wang, P. (2024). Ecotoxicity of hexavalent chromium [Cr(VI)] in soil presents predominate threats to agricultural production with the increase of soil Cr contamination. *Journal of Hazardous Materials, 476*, 135091. [https://doi.org/10.1016/j.jhazmat.2024.135091](https://doi.org/10.1016/j.jhazmat.2024.135091?utm_source=chatgpt.com)

Kokkinos, E., Kotsali, V., Tzamos, E., & Zouboulis, A. (2024). Acid mine drainage neutralization by ultrabasic rocks: A chromite mining tailings evaluation case study. *Sustainability, 16*, 8967. [https://doi.org/10.3390/su16208967](https://doi.org/10.3390/su16208967?utm_source=chatgpt.com)

Kushkevych, I., Dordević, D., & Vítězová, M. (2019). Toxicity of hydrogen sulfide toward sulfate-reducing bacteria Desulfovibrio piger Vib-7. *Archives of Microbiology, 201*, 389–397. [https://doi.org/10.1007/s00203-019-01625-z](https://doi.org/10.1007/s00203-019-01625-z?utm_source=chatgpt.com)

Moon, D.H., Wazne, M., Jagupilla, S.C., Christodoulatos, C., Kim, M.G., & Koutsospyros, A. (2008). Particle size and pH effects on remediation of chromite ore processing residue using calcium polysulfide (CaS5). *Science of the Total Environment, 399*, 2–10. [https://doi.org/10.1016/j.scitotenv.2008.03.040](https://doi.org/10.1016/j.scitotenv.2008.03.040?utm_source=chatgpt.com)

Rashid, A., Ayub, M., Ullah, Z., Ali, A., Sardar, T., Iqbal, J., Gao, X., Bundschuh, J., Li, C., Khattak, S.A., Ali, L., El-Serehy, H.A., Kaushik, P., & Khan, S. (2023). Groundwater quality, health risk assessment, and source distribution of heavy metals contamination around chromite mines: Application of GIS, sustainable groundwater management, geostatistics, PCAMLR, and PMF receptor model. *International Journal of Environmental Research and Public Health, 20*, 2113. [https://doi.org/10.3390/ijerph20032113](https://doi.org/10.3390/ijerph20032113?utm_source=chatgpt.com)

Telfeyan, K., Migdisov, A.A., Pandey, S., Vesselinov, V.V., & Reimus, P.W. (2019). Long-term stability of dithionite in alkaline anaerobic aqueous solution. *Applied Geochemistry, 101*, 160–169. [https://doi.org/10.1016/j.apgeochem.2018.12.015](https://doi.org/10.1016/j.apgeochem.2018.12.015?utm_source=chatgpt.com)

Verma, Y., Verma, A., Bhaskaralingam, A., Dhiman, P., Wang, T., Kumar, A., & Sharma, G. (2025). Application of zero-valent iron and its derivatives in the removal of toxic metal ions from groundwater. *Water, 17*, 1524. [https://doi.org/10.3390/w17101524](https://doi.org/10.3390/w17101524?utm_source=chatgpt.com)

Villalobos-Aragón, A., Ellis, A.S., Armienta, M.A., & Morton-Bermea, O. (2012). Geochemistry and Cr stable isotopes of Cr-contaminated groundwater in León valley, Guanajuato, México. *Applied Geochemistry, 27*, 1783–1794. [https://doi.org/10.1016/j.apgeochem.2012.02.013](https://doi.org/10.1016/j.apgeochem.2012.02.013?utm_source=chatgpt.com)

Wei, Y., Xu, X., Zhao, L., Chen, X., Qiu, H., & Gao, B. (2021). Migration and transformation of chromium in unsaturated soil during groundwater table fluctuations induced by rainfall. *Journal of Hazardous Materials, 416*, 126229. [https://doi.org/10.1016/j.jhazmat.2021.126229](https://doi.org/10.1016/j.jhazmat.2021.126229?utm_source=chatgpt.com)
